# The Extent and Nature of Functional Limitations According to the Health Assessment Questionnaire Disability Index in Patients with Rheumatoid Arthritis and Severe Functional Disability

**DOI:** 10.3390/jcm13020379

**Published:** 2024-01-10

**Authors:** Max M. H. Teuwen, Maria A. T. van Wissen, Wilfred F. Peter, Dirkjan van Schaardenburg, Cornelia H. M. van den Ende, Maaike G. J. Gademan, Salima F. E. van Weely

**Affiliations:** 1 Department of Orthopaedics, Rehabilitation and Physical Therapy, Leiden University Medical Center, 2300 RC Leiden, The Netherlands; m.m.h.teuwen@lumc.nl (M.M.H.T.);; 2 Center for Rehabilitation and Rheumatology, Reade, 1056 AA Amsterdam, The Netherlands; 3 Department of Rheumatology, Radboud UMC, 6500 GM Nijmegen, The Netherlands; 4 Department of Research, Sint Maartenskliniek, 6500 GM Nijmegen, The Netherlands; 5 Department of Clinical Epidemiology, Leiden University Medical Center, 2300 RC Leiden, The Netherlands

**Keywords:** rheumatoid arthritis, functional disability, HAQ-DI, rehabilitation, patient-reported outcomes

## Abstract

Background: For a subgroup of people with rheumatoid arthritis (RA) and severe disability, insight into their limitations is crucial for adequate treatment. Aim: To describe the extent and nature of functional limitations in people with RA and severe disability and to explore the associations of the extent of the functional limitations with patient characteristics, disease characteristics, and outcome measures. Methods: Baseline data of 215 participants in an RCT on the (cost-)effectiveness of longstanding physiotherapy were used. Functional limitations were assessed with the Health Assessment Questionnaire Disability Index (HAQ-DI). The total HAQ-DI including eight domain scores were calculated. Associations between high HAQ-DI scores (≥2, yes/no) and other variables were examined using the Student’s *t*-test or Chi-squared test where appropriate. Results: The participants (90% women, age 58.8 ± 12.8 years) had a mean HAQ-DI score of 1.7 ± 0.5. The majority (56%) showed a moderate-to-severe disability in all domains. Higher HAQ-DI scores seemed to be associated with advanced age, longer disease duration, unemployment, joint replacements, and outcomes for daily functioning and physical quality of life, but not with measures of disease activity. Conclusions: Our findings indicate that a comprehensive assessment of all areas of daily activities in this subgroup is necessary in order to provide appropriate (non-)pharmacological care.

## 1. Introduction

Rheumatoid arthritis (RA) is an autoimmune disease that causes inflammation and the progressive destruction of the joints affecting approximately 0.5–1% of the general population [1,2,3]. It can lead to pain, fatigue, and functional disability with a considerable impact on quality of life (QoL) [3,4,5]. Pharmacological treatment options have greatly improved in recent decades, leading to an overall better level of functioning and QoL [6]. Despite the availability of these effective treatments, there is a subgroup of people with RA who have considerable functional limitations due to persistently high disease activity and a proportion of people in this subgroup may be classified as difficult-to-treat RA (D2T) [7,8]. Moreover, existing joint destruction, deformities, or the presence of comorbidities can also contribute to functional disability [9]. This can affect people’s ability to perform even simple everyday activities [10,11,12,13] and may mean that they require support with non-pharmacological treatment modalities such as physiotherapy, occupational therapy, or rehabilitation [8,14]. More insight into both the extent and nature of the functional disability is important to provide adequate treatment that meets the (therapeutic) needs of this specific subgroup and direct (non-)pharmacological interventions to the right domains thereby improving the disability and QoL.

To our knowledge, there is a paucity of studies examining the nature of functional disability in general and especially in the subgroup of people with RA and severe disability. One study assessed the limitations in physical functioning in people with RA eligible for rehabilitation treatment and thus likely to have considerable disability [15]. In that study, executed in four countries, the content of rehabilitation treatment goals was analyzed using the International Classification of functioning disability and Health (ICF) as a reference [15]. It was shown that most limitations (44%) were observed in the domain “Activities and Participation”, with the top 3 pertaining to “Learning and applying knowledge” (d1), “Mobility” (d4), and “Self-care” (d5) [15]. A recent, comparable study by our research group linked the prioritized functional limitations, as measured with the Patient Specific Complaints instrument (PSC) [16] into ICF categories. For this study, people with RA who participated in a randomized controlled trial (RCT) of long-term exercise therapy were included. From that study, it was concluded that limitations in activities were most frequently seen in “Walking”, “Changing basic body position”, “Grasping”, and “Stair climbing”. However, both studies used a method to assess and prioritize personalized treatment goals, rather than a fixed set of activities. This complicates the interpretation and comparability of problems within groups and between individuals. The most frequently used patient-reported outcome measure to assess functional ability in people with RA in both research and clinical settings is the Health Assessment Questionnaire Disability Index (HAQ-DI) [17,18]. The HAQ-DI assesses an individual’s abilities over the past week using their usual equipment. The HAQ-DI is mainly used to quantify (changes in) the degree of disability severity and focuses on limitations in activities, taking into account some aspects of the patient’s physical environment [19]. It was applied and validated in patients with a wide variety of rheumatic diseases, including RA [17,18]. It is sensitive to change and is a good predictor of future disability [9,20], sustained remission in the course of RA [21], and provides decision support where there is a need for multidisciplinary interventions [22]. However, these papers lack details on the nature of the limitations and did not specifically report on individuals within the RA population who were experiencing severe functional disability. 

Moreover, within the broader RA population, the HAQ-DI exhibits a robust correlation with various measures of physical functioning and QoL, including the Patient-Reported Outcomes Measurement Information System Physical Function 10 (PROMIS PF-10) [23] and the 36-Item Short-Form Health Survey Physical Component Summary Scale (SF-36 PCS) [24]. 

While functional disability is known to be linked to disease activity [9,25,26], it is imperative to recognize that it can also be influenced by other factors such as joint damage, deformities, complications arising from the disease or its treatment, and/or comorbidities [9,26]. 

The subgroup of individuals with RA and severe functional disability forms a distinctive cohort, potentially requiring unique support from various healthcare professionals. The absence of exploration into this specific subgroup raises critical questions about the specific challenges they encounter in their daily lives and the potential impact on their overall well-being. Gaining insight into the severity and nature of functional limitations through the HAQ-DI, along with exploring associations with patient or disease characteristics and other questionnaires, could contribute to refining healthcare strategies. This approach aims to foster a more targeted and personalized approach to managing severe functional disability in individuals with RA.

Therefore, the objective of our study was twofold: first, to describe the extent and nature of functional limitations in people with RA using the HAQ-DI, and second, to explore which patient characteristics, disease characteristics, and/or other measures of physical functioning and QoL are associated with a higher HAQ-DI score within this subgroup.

## 2. Materials and Methods

### 2.1. Study Design

This is a secondary analysis of baseline data from an RCT comparing the (cost-)effectiveness of longstanding exercise therapy to usual care in people with RA and severe functional disability. A detailed description of the study methods including inclusion and exclusion criteria, assessments, study procedures, and intervention description for this RCT is published elsewhere [27]. The trial was approved by the Medical Ethical Committee Leiden-Den Haag-Delft (METC LDD; L-EXTRA: NL69866.058.19) and registered in the International Clinical Trials Registry Platform (ICTRP): (Longstanding EXercise Therapy in patients with Rheumatoid Arthritis; L-EXTRA; NL8235). The study was conducted in accordance with the principles of the Declaration of Helsinki (2013) [28]. All participants gave written informed consent before entering the study. 

### 2.2. Participants

Participants were recruited via various social media channels and 50 rheumatology outpatient departments spread across all provinces of the Netherlands. Adults (aged ≥ 18 years), with a clinical diagnosis of RA made by a rheumatologist and self-perceived severe limitations in functioning involving self-care (e.g., dressing and washing), and/or transfers (e.g., getting in and out of bed, rising from a chair or using the toilet), and/or mobility indoors or outdoors were included. Limitations had to be directly or indirectly related to the rheumatic condition, e.g., caused by persisting or progressive disease activity despite optimal medical treatment and/or severe joint damage and/or deformities and/or severe comorbidity, e.g., pulmonary or cardiovascular disease. Furthermore, it was judged that their functional limitations were unlikely to improve or resolve with a brief exercise therapy intervention, as assessed by two experienced physical therapists involved in the research team.

### 2.3. Assessments

For the current study, we used the following baseline assessments: the baseline questionnaire, which was filled out online by all participants, and the baseline the 6 min walk test (6MWT). The 6MWT was assessed by a researcher (M.M.H.T. or M.A.T.v.W. following a standardized protocol published in the study protocol [27]). Reminders were given by mail and telephone. Disease characteristics were retrieved via the treating rheumatologist. The following characteristics were used: socio-demographics (gender, age, body mass index, single person household, education level, work status (in participants aged ≤66 years), disease characteristics (symptom duration, disease duration, fulfilment of D2T RA criteria [7], rheumatoid factor (RF-positive), anti-citrullinated protein antibodies (ACPA positive), Disease Activity (Disease Activity Score 28 joints, DAS-28) [29,30], current medication), medical history (smoking status, number of co-morbidities, number of joint replacement surgeries), measures of physical functioning (Patient-Reported Outcome Measurement Information System Physical Function 10-Item Short Form (PROMIS PF-10, range 13.5 to 61.9 with higher scores corresponding with better physical functioning) [31,32,33], the HAQ-DI [17,18]), measures of QoL (36-Item Short-Form Health Survey Mental and Physical Component Summary Scale (SF-36 MCS/PCS, range 0–100, worst–best) [34,35]), measure of performance-based physical functioning (6MWT, distance walked in meters). 

#### HAQ-DI and Functional Disability

The HAQ-DI was used to assess the extent and nature of functional disability [17,18]. The HAQ-DI reflects problems in activities of daily living and consists of 20 items divided over eight domains (Dressing and grooming, Arising, Eating, Walking, Personal hygiene, Reaching, Gripping and Usual activities). Each item is scored on a 4-point scale ranging from 0 to 3 (0 = without ANY difficulty 1 = with SOME difficulty, 2 = with MUCH difficulty, 3 = UNABLE to do), with the total HAQ-DI score ranging from 0 to 3 (no disability to very severe disability) [36]. To determine the participants’ functional disability, domain scores and the total HAQ-DI score were calculated with the correction for the use of assistive devices. The use of aids or devices or physical assistance increases a score of 0 or 1 to a 2 to more accurately represent underlying disability; scores of 3 are not modified. Scores of 0 to 1 generally represent mild-to-moderate difficulty, 1 to 2 represent moderate-to-severe disability, and 2 to 3 indicate severe-to-very-severe disability [17]. A HAQ-DI score of <0.5 is often used in studies to describe normative physical function [5].

### 2.4. Statistical Analyses

The data are presented as mean (standard deviation (SD)) and number and percentage (*n*, %) as appropriate. To assess the extent of functional disability the HAQ-DI score and domain scores (mean, SD) were calculated. To get more insight into the nature of functional disability within each domain the percentages of participants reporting no/some/much difficulty of inability to perform the activities was calculated and the domain with the highest percentage of participants with a maximum score of 3 was identified. In addition, for each participant, the number of domains with a score ≥ 1 was determined. 

To explore potential associations with patient characteristics, disease characteristics, and other baseline questionnaire variables, we categorized patients into two groups based on their HAQ-DI scores, distinguishing between high and lower scores. We arbitrarily established, on the basis of consensus among authors, a cut-off point at a HAQ-DI score < 2 (mild-to-moderate) and a HAQ-DI score ≥ 2 (severe-to-very-severe). Subsequently, we conducted unpaired *t*-tests (including mean difference and 95% Confidence Interval (CI)) for continuous data, Pearson’s Chi-squared tests and calculated the odds ratio (OR) and 95% CI for dichotomous data to assess potential associations. Considering the explorative nature of these analyses we did not perform a formal sample size calculation.

Statistical analyses were performed in IBM SPSS Statistics version 25.0 (Released 2017, Armonk, NY, USA: IBM Corp.).

## 3. Results

### 3.1. Baseline Characteristics

The baseline characteristics of the study population are summarized in Table 1. In total, data from 215 participants with RA were used who were mainly women (*n* = 194; 90%) with a mean age of 58.8 (SD ± 12.8) years and a mean disease duration of 18.8 (±13.0) years. Among the working-age participants (*n* = 154, 72%), the majority were not employed due to health problems (*n* = 61, 40%). Nearly half of the study population met the D2T RA criteria [7] (*n* = 90, 47%), and 37% (*n* = 80) had undergone one or more joint replacement surgeries, and the majority had three or more comorbidities (*n* = 154/213, 72%). The mean scores for the PROMIS PF-10 (33.9 ± 5.1) and SF-36 PCS scores (29.5 ± 7.8) are indicative of considerable disability.

### 3.2. Extent and Nature of Functional Disability

Data showed considerable disability within the study population, as indicated by a mean HAQ-DI score of 1.7 (SD ± 0.5). In addition, the majority (*n* = 200, 93%) had a HAQ-DI score of ≥1, indicating moderate-to-severe functional disability.

**Figure 1:**
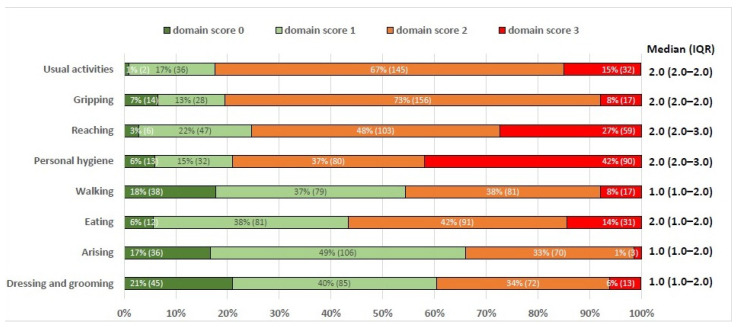
The percentages and number of participants with domain scores ranging from 0 to 3 for each domain and mean HAQ-DI domain scores (*n* = 215).

Figure 1 shows the median HAQ-DI domain scores. All median scores were ≥1, ranging from 1.0 to 2.0 with a minimum score of 0 and a maximum score of 3. It also shows the percentage and number of participants with domain scores 0, 1, 2, or 3 for all eight domains. Most severe limitations were observed in the domains of Usual Activities, Gripping, Reaching and Personal hygiene, with 75% or more of the study population showing a domain score of 2 or 3, indicating severe disability. The highest percentage of participants with a domain score of 3 was seen in the domain of Personal hygiene (42%, *n* = 90).

**Figure 2:**
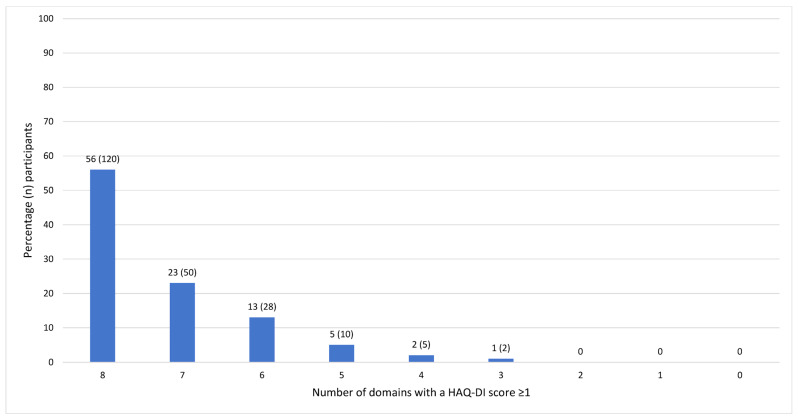
Frequencies of the total number of domains with a HAQ-DI domain score of ≥1 (percentage and number of participants, *n* = 215).

Regarding the number of domains with a score of ≥1, the frequencies are shown in Figure 2 and the interquartile range was 7–8 (range 3–8). All participants showed to be limited in at least three domains, with the majority of the participants having a domain score ≥1 in all eight (56%, *n* = 120) or seven domains (23%, *n* = 50). In addition, 13% (*n* = 28) showed limitations in six domains and only the minority (*n* = 17, 8%) in five or fewer domains, indicating that within this population of people with RA, functional disability is present in most domains. 

**Figure 3:**
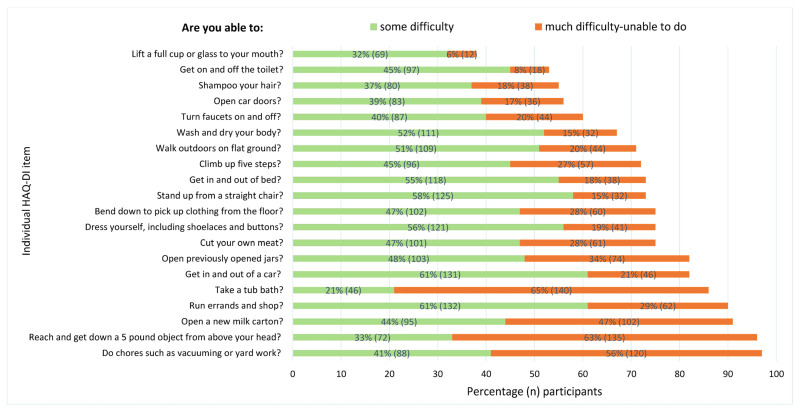
Frequencies of participants experiencing at least some difficulty (score ≥ 1) per individual item on the HAQ-DI (percentage and number of participants, *n* = 215).

Figure 3 shows the distribution of patients reporting a score of ≥1 (indicating some difficulties or worse) for individual items on the HAQ-DI. While scores varied, most items showed a percentage of at least 53% of patients reporting a score ≥1, barring one exception: “Lift a full cup or glass to your mouth”. Conversely, the most restrictive item, “Do chores such as vacuuming or yard work”, has a notably higher prevalence, with 97% of patients reporting a score of ≥1.

For items most frequently reported with a score of 2 or 3 (indicating much difficulty or an inability to perform), three stand out across different domains. In the Usual activities domain, “Do chores such as vacuuming or yard work” is particularly challenging for 56% of patients. In the Reaching domain, “Reach and get down a 5-pound object from above your head” proves to be of substantial difficulty for 63% of patients. Similarly, in the Personal hygiene domain, “Take a tub bath” is notably challenging, with 65% of patients scoring 2 or higher.

In Table 2, characteristics stratified for individuals with HAQ-DI scores < 2 and those with HAQ-DI scores ≥ 2 are presented including their associations. Advanced age (mean difference −3.9 [95% CI: −7.8, −0.02]), longer disease duration (mean difference −8.3 [95% CI: −13.0, −3.7]), unemployment (OR: 0.21 [95% CI: 0.07, 0.6]), and the presence of one or more joint replacements (OR: 2.1 [95% CI: 1.1, 3.8]) were associated with HAQ-DI scores ≥ 2. Additionally, on average, patients with higher HAQ-DI scores ≥ 2 had a lower PROMIS-PF-10 score (mean difference 7.6 [95% CI: 6.1, 9.0]), a reduced SF-36 PCS (mean difference [95% CI: 6.1 [4.0, 8.1]), and a shorter distance covered in the 6 min walk test (mean difference 98 [95% CI: 72, 124]). No significant differences were observed in any of the other characteristics between the two HAQ-DI score groups.

## 4. Discussion

In a subgroup of people with RA, the extent and nature of self-reported functional disability was assessed. The complexity of their condition was illustrated by the considerable proportions of people with multiple comorbidities and fulfilling the criteria for D2T RA [7]. The majority showed to have considerable limitations in almost all the domains of the HAQ-DI. Limitations were most prevalent in the domains of Usual activities, Personal hygiene and Reaching. The extent and nature of the reported disability may indicate the need for support from multiple healthcare professionals meeting the specific needs of this subgroup. Higher HAQ-DI scores seemed to be associated with advanced age, longer disease duration, unemployment, joint replacements, and outcomes for daily functioning and physical QoL, but not with measures of disease activity.

There is a paucity of studies assessing the extent and nature of functional limitations in people with RA, especially for this specific subgroup. Participants in this study showed moderate-to-severe disability, reflected in a mean HAQ-DI of 1.7 (0.5). This finding is partly consistent with an RCT in which people with RA who were hospitalized for multidisciplinary treatment in a rheumatology clinic due to active RA and loss of functional ability showed HAQ-DI values of 1.8 (0.8) and 1.7 (0.6), respectively [37]. However, this study was conducted more than twenty years ago. Furthermore, the HAQ-DI value we observed was significantly higher than the HAQ score of 0.9 (0.5) observed in a recent study, in a subpopulation of D2T RA patients consecutively selected from a hospital population [38]. A lower HAQ-DI score of 1.27 was also observed in a population with generalized osteoarthritis referred for multidisciplinary treatment [39]. This shows that our study population has a high HAQ-DI score, indicating a high clinical burden, although they were treated in primary care and not in a multidisciplinary rehabilitation setting. 

In a study by Meesters et al. [15], in which treatment goals of an RA population in rehabilitation care were linked to the ICF, most limitations in functioning were seen within the chapters “Learning and applying knowledge” (d1), “General tasks and demands” (d2), “Communication” (d3), “Mobility” (d4) and “Self-care” (d5). However, not all these chapters can be linked to the domains of the HAQ-DI. Our findings are partly in line with a previous study from our study group in the same population. Here, the treatment goals elicited and prioritized with the PSC were linked to the ICF, in which “Walking”, “Changing body position” and “Grasping” were the most frequently identified ICF codes [16]. However, the methods for assessing disability using the PSC cannot be compared to a predefined list of activities as defined in the HAQ-DI.

When assessing the severity of the HAQ-DI, one other study also focused on the individual items [40]. This particular study involved an RA population characterized by a relatively favorable health status and absence of serious comorbidities, leading to lower average scores per individual item compared to our specific population [40]. Nevertheless, noteworthy similarities were observed in the items exhibiting the highest disability scores. The three items displaying the highest scores mirrored our study, encompassing challenges related to “Doing chores such as vacuuming or yard work”, “Reach and get down a 5-pound object”, and “Open a new carton of milk” [40].

The substantial number of participants facing challenges in performing activities of daily living within the domains of Personal hygiene, Reaching, and Usual activities primarily resulted from limitations in a single key item. Within each respective domain, these key items were: “Taking a tub bath”, “Doing chores such as vacuuming or yard work”, or “Reaching and getting down a 5-pound object from above your head”. Although these specific items may require extra attention, it should also be noted that the majority of participants reported at least some difficulty in all other individual items. The only exception here was “Lifting a full cup or glass to your mouth”. This broad range of challenges underscores the overall disability experienced by this subgroup, wherein the joints of the upper, lower, or both upper and lower extremities may be affected. 

Nearly two-thirds of participants encountered difficulties in a seemingly straightforward lower limb activity, such as “Walking outdoors on flat ground”. Conversely, over 90% face challenges in upper extremity activities like opening a milk carton and reaching. These findings strongly indicate that both upper and lower extremity functioning are compromised in the majority of patients.

Furthermore, it is noteworthy that tasks such as getting on and off the toilet may present a complex challenge for this subgroup. This complexity arises from the multifaceted nature of the task, encompassing actions such as transferring, wiping, and washing hands, all of which necessitate adequate functioning of both upper and lower extremities. Even with the use of aids such as a raised toilet or grab rails, achieving independence in performing this task remains elusive for many. Clinicians and health professionals should be attuned to this complexity, actively acquiring about the specific difficulties individuals encounter in these activities and exploring viable options to enhance independence. Understanding the nuanced challenges within these daily tasks is paramount for tailored interventions and support.

Remarkably, we did not find a difference between the patients with HAQ-DI scores < 2 and those with HAQ-DI scores ≥ 2 in measures of disease activity as several studies demonstrated this association [9,25,26]. This discrepancy might be explained by the relatively high proportion of individuals in our study who could be classified as D2T. It is conceivable that the variation in disease activity among individuals in our study was too small to detect an association between functional disability and disease activity.

This study has a number of limitations. The present findings are restricted to patients with severe functional disability included for an RCT on long-term exercise therapy and thus concerned a selected population that might not be generalizable to the total RA population. People with a relatively positive attitude towards exercise therapy may have been overrepresented. Furthermore, although we endorse the ‘Sex and Gender Equity in Research—SAGER—guidelines’ no gender analyses were conducted, as our population consisted almost entirely of women. It is known that women are in general more willing to participate in research than men [41] and this could have influenced the results. In well-treated people with RA, HAQ-DI scores between men and women are similar [42]. However, with increasing disease duration and severity, people with RA tend to overestimate their functional ability, while RA patients in the early stages of the disease tend to underestimate their functional ability [37,42]. In an RA population with moderate-to-severe disability, men tended to overestimate their functional ability considerably more than females [43]. Several studies have argued that the interpretation of what people perceive as difficult changes over time and is influenced by consciously and unconsciously made adjustments in the performance of activities in daily living. Since functional disability in general may increase over the disease course, attention to the level and nature of perceived limitations is of paramount importance for clinical practice. 

Additionally, due to the predominant representation of females, the advanced age of the participants, and the prevalence of a substantial number of comorbidities within the cohort we faced limitations in conducting meaningful subgroup analyses, which could have offered more nuanced insights into variations in functional disability across specific patient or disease characteristics. 

Unfortunately, we did not collect data on the location or number of the affected joints, impeding in-depth analysis about the association of the location or number of affected joints and reported limitations in activities on the HAQ-DI. The shoulder, knee, and elbow joints are known to account for approximately 70% of the total contribution to the HAQ [44], underscoring the involvement of both the upper and lower extremities. Subsequent research should aim to address this gap in information.

Furthermore, it is imperative to underscore that our study relies on cross-sectional data, inherently restricting our ability to infer changes over time. Also, by conducting multiple statistical tests, potential pitfalls are introduced, including an increased risk of Type I errors and the potential for false positive findings. In addition, the absence of a dedicated power analysis underscores the importance of approaching these results with caution. 

A strength is that the present study was nationwide and includes a relatively high number of subjects which could also facilitate the generalizability of the results. Furthermore, this is the first study documenting the extent and nature of functional limitations in eight domains of the HAQ for this subgroup. Future research in other subgroups of RA people could provide a more comprehensive insight into the extent to which people with RA experience functional limitations, despite the availability of advanced pharmacological treatment options.

## 5. Conclusions

In conclusion, in this RA subgroup with self-reported severe disability, there are profound functional limitations in all domains, with limitations in the domains of Personal hygiene and Reaching being the most prevalent. Therefore, a comprehensive assessment of all areas of daily activities in this RA subgroup is necessary for daily clinical practice. Insight into the domains of functional disability could lead physicians and health professionals to target relevant and modifiable factors to regain and maintain function and prevent disability. In addition, the extent and nature of the reported disability may indicate the need for non-pharmacological care that meets the specific needs of this subgroup. Higher HAQ-DI scores seemed to be associated with advanced age, longer disease duration, unemployment, joint replacements, and outcomes for daily functioning and physical QoL, but not with measures of disease activity. However, additional research is imperative to validate and substantiate these identified associations.

## Figures and Tables

**Table 1:** Baseline characteristics of participants with RA and severe disability (*n* = 215).

Female, *n* (%)	194 (90)
Age in years, mean (SD)	58.8 (12.8)
BMI (kg/m^2^), mean (SD)	27.6 (6.0)
Single-person household, *n* (%)	67 (31)
Higher Education, *n* (%)	62 (29)
Work status, *n* (%)	
≤66 years old, *n* (%)	154 (72)
Paid job, *n* (%)	45 (29)
No job, health problems, *n* (%)	61 (40)
No job, other reasons, *n* (%)	48 (31)
Self-reported symptom duration (years), mean (SD)	21.6 (13.3)
Disease duration (years), Mean (SD)	18.8 (13.0) (*n* = 193)
Difficult-to-treat RA criteria ^a^, *n* (%)	90 (47) (*n* = 191)
RF positive, *n* (%)	127 (68) (*n* = 187)
ACPA positive, *n* (%)	113 (61) (*n* = 184)
DAS-28 ^b^, mean (SD)	3.1 (1.3) (*n* = 159)
Current medication use ^c^, *n* (%)	
Any DMARD	149 (69)
bDMARD	114 (77)
tsDMARD	12 (8)
csDMARD	86 (58)
NSAIDs	98 (46)
Glucocorticoids Oral	51 (24)
Glucocorticoids Injection intra-muscular/intra-articular	31 (14)
No RA treatment-related medication	10 (9)
Number of comorbidities, *n* (%)	(*n* = 213)
0	8 (4)
1–2	51 (24)
3–4	72 (33)
≥5	82 (39)
Joint replacement surgeries ≥1, *n* (%)	80 (37)
PROMIS PF-10, mean (SD)	33.9 (5.1)
SF-36 MCS, mean (SD)	46.7 (12.4)
SF-36 PCS, mean (SD)	29.5 (7.8)
HAQ-DI mean (SD)	1.7 (0.5)
6MWT distance (meters), mean (SD)	305 (96) (*n* = 213)

Abbreviations and explanatory: ACPA, anti-citrullinated protein antibodies; BMI, Body Mass Index; DAS-28, Disease Activity Score; DMARDs, Disease-Modifying Antirheumatic Drugs; HAQ-DI, Health Assessment Questionnaire Disability Index; Higher education, Bachelor or Master at University (of Applied Sciences), and doctoral degree program at research universities; PROMIS PF-10, Patient Reported Outcomes Measurement Information System Physical Functioning-10; RF, Rheumatoid Factor; bDMARDS, biological Disease-Modifying Antirheumatic Drugs; tsDMARD, targeted synthetic Disease-Modifying Antirheumatic Drugs; csDMARD conventional synthetic Disease-Modifying Antirheumatic Drugs; SF-36 MCS/PCS, 36-Item Short-Form Health Survey Mental/Physical Component Summary Scale; 6MWT, 6-min walk test. ^a^ Difficult-to-treat RA definition based on Nagy et al. [7]. ^b^ DAS-28 score is based on the ESR and if the DAS-28 score was based on the CRP score the following calculation was used: DAS28-ESR = 3.3928 × Ln (DAS-28-CRP) + 0.0254 [30]. ^c^ Multiple answers possible.

**Table 2:** Comparison of patient and disease characteristics between HAQ-DI scores < 2 and HAQ-DI scores ≥ 2.

	HAQ-DI < 2 (*n* = 157)	HAQ-DI ≥ 2 (*n* = 58)	*p*-Value	Mean Difference [95% CI] ^c^	OR [95% CI] ^d^
Female, *n* (%)	140 (89)	54 (93)	*p* = 0.39	NA	1.6 [0.5, 5.1]
Age in years, mean (SD)	57.7 (12.7)	61.6 (13.0)	*p* = 0.049	−3.9 [−7.8, −0.02]	NA
BMI (kg/m^2^), mean (SD)	27.4 (5.7)	28.1 (6.8)	*p* = 0.44	−0.8 [−2.8, 1.2]	NA
Single-person household, *n* (%)	49 (31)	18 (31)	*p* = 0.98	NA	1.0 [0.5, 1.9]
Higher Education, *n* (%)	50 (32)	12 (21)	*p* = 0.11	NA	0.6 [0.3, 1.2]
Works status: ≤66 years old (*n* = 154)					
Having a Paid job, *n* (%)	41 (26)	4 (7)	*p* = 0.002	NA	0.21 [0.07, 0.6]
Disease duration (years), Mean (SD)	16.6 (11.2) (*n* = 141)	24.9 (15.5) (*n* = 52)	*p* < 0.001	−8.3 [−13.0, −3.7]	NA
Difficult-to-treat RA criteria ^a^, *n* (%)	34 (42) (*n* = 81)	56 (49) (*n* = 110)	*p* = 0.26	NA	1.45 [0.8, 2.8]
RF positive, *n* (%)	48 (62) (*n* = 78)	79 (73) (*n* = 109)	*p* = 0.63	NA	1.2 [0.6, 2.4]
ACPA positive, *n* (%)	51 (64) (*n* = 80)	62 (60) (*n* = 104)	*p* = 0.12	NA	0.6 [0.3, 1.1]
DAS-28 ^b^, mean (SD)	3.1 (1.3) (*n* = 117)	3.1 (1.4) (*n* = 42)	*p* = 0.99	−0.004 [−0.5, 0.5]	NA
Number of comorbidities ≥2, *n* (%)	75 (84) (*n* = 89)	110 (89) (*n* = 124)	*p* = 0.25	NA	1.8 [0.7, 5.0]
Joint replacement surgeries ≥1, *n* (%)	18 (20)	62 (50)	*p* = 0.018	NA	2.1 [1.1, 3.8]
PROMIS PF-10, mean (SD)	35.9 (3.4)	28.4 (5.0)	*p* < 0.001	7.6 [6.1, 9.0]	NA
SF-36 PCS, mean (SD)	31.1 (7.8)	25.1 (6.2)	*p* < 0.001	6.1 [4.0, 8.1]	NA
SF-36 MCS, mean (SD)	47.4 (12.2)	44.7 (12.8)	*p* = 0.17	2.6 [−1.1, 6.4]	NA
6MWT distance (meters), mean (SD)	332 (82) (*n* = 155)	234 (94)	*p* < 0.001	98 [72, 124]	NA

Abbreviations and explanatory: ACPA, anti-citrullinated protein antibodies; BMI, Body Mass Index; DAS-28, Disease Activity Score; Higher education, Bachelor or Master at University (of Applied Sciences); and doctoral degree program at research universities; PROMIS PF-10, Patient Reported Outcomes Measurement Information System Physical Functioning-10; RF, Rheumatoid Factor; SF-36 MCS/PCS, 36-Item Short-Form Health Survey Mental/Physical Component Summary Scale; 6MWT; 6-min walk test. ^a^ Difficult-to-treat RA definition based on Nagy et al. [7]. ^b^ DAS-28 score is based on the ESR and if the DAS-28 score was based on the CRP score the following calculation was used: DAS28-ESR = 3.3928 ×Ln (DAS-28-CRP) + 0.0254 [30]. ^c^ Independent samples *t*-test calculating the mean difference and 95% confidence interval. ^d^ Odds ratio and 95% confidence interval.

## Data Availability

The data presented in this study are available on request from the corresponding author. The data are not publicly available due to privacy reasons.
